# An Ab Initio Investigation of the Geometries and Binding Strengths of Tetrel-, Pnictogen-, and Chalcogen-Bonded Complexes of CO_2_, N_2_O, and CS_2_ with Simple Lewis Bases: Some Generalizations

**DOI:** 10.3390/molecules23092250

**Published:** 2018-09-04

**Authors:** Ibon Alkorta, Anthony C. Legon

**Affiliations:** 1 Instituto de Química Médica (IQM-CSIC), Juan de la Cierva, 3, E-28006 Madrid, Spain; 2 School of Chemistry, University of Bristol, Cantock’s Close, Bristol BS8 1TS, UK

**Keywords:** intermolecular force constants, dissociation energies, CCSD(T)/aug-cc-pVTZ calculations, non-covalent bonds

## Abstract

Geometries, equilibrium dissociation energies (*D*_e_), and intermolecular stretching, quadratic force constants (*k*_σ_) are presented for the complexes B⋯CO_2_, B⋯N_2_O, and B⋯CS_2_, where B is one of the following Lewis bases: CO, HCCH, H_2_S, HCN, H_2_O, PH_3_, and NH_3_. The geometries and force constants were calculated at the CCSD(T)/aug-cc-pVTZ level of theory, while generation of *D*_e_ employed the CCSD(T)/CBS complete basis-set extrapolation. The non-covalent, intermolecular bond in the B⋯CO_2_ complexes involves the interaction of the electrophilic region around the C atom of CO_2_ (as revealed by the molecular electrostatic surface potential (MESP) of CO_2_) with non-bonding or π-bonding electron pairs of B. The conclusions for the B⋯N_2_O series are similar, but with small geometrical distortions that can be rationalized in terms of secondary interactions. The B⋯CS_2_ series exhibits a different type of geometry that can be interpreted in terms of the interaction of the electrophilic region near one of the S atoms and centered on the C_∞_ axis of CS_2_ (as revealed by the MESP) with the n-pairs or π-pairs of B. The tetrel, pnictogen, and chalcogen bonds so established in B⋯CO_2_, B⋯N_2_O, and B⋯CS_2_, respectively, are rationalized in terms of some simple, electrostatically based rules previously enunciated for hydrogen- and halogen-bonded complexes, B⋯HX and B⋯XY. It is also shown that the dissociation energy *D*_e_ is directly proportional to the force constant *k*_σ_, with a constant of proportionality identical within experimental error to that found previously for many B⋯HX and B⋯XY complexes.

## 1. Introduction

Investigation, both experimentally and theoretically, of non-covalent interactions among molecules is a topic of rapidly increasing interest. The hydrogen bond, known for almost a century, is of fundamental importance in chemistry and biology. The halogen bond is a weak interaction, in which interest within both disciplines grew rapidly in the last two decades. Modern definitions of the hydrogen bond [1] and the halogen bond [2], made under the auspices of the International Union of Pure and Applied Chemistry (IUPAC), arose naturally from the increased activity. Tetrel bonds, pnictogen bonds, and chalcogen bonds, close relatives of hydrogen and halogen bonds, were recognized as weak, non-covalent interactions in both the gas phase [3] and condensed phase [4] for several decades, but were named only in 2013 [5], 2011 [6], and 2009 [7], respectively. A task group set up by the IUPAC is currently working on the definitions of these three, newly named interactions (see: https://iupac.org/projects/project-details/?project_nr=2016-001-2-300).

It is now widely accepted [2,3,8] that each of these non-covalent bonds arises mainly from the interaction of an electrophilic region associated with an atom of the element E (where E is hydrogen, a halogen, or an element of group 14, 15, or 16) with the nucleophilic region (e.g., a non-bonding or π-bonding electron pair) in another molecule or the same molecule. Electrophilic and nucleophilic regions can be identified via the electrostatic potential near to the appropriate regions of the molecules [9]. A convenient modern and readily available way of identifying such regions is the molecular electrostatic surface potential (MESP), which is the potential energy of a non-perturbing, unit-positive point charge at the iso-surface on which the electron density is constant [10], and it is usually expressed as 0.00*n* e/bohr^3^ (*n* = 2 here).

**Figure 1:**
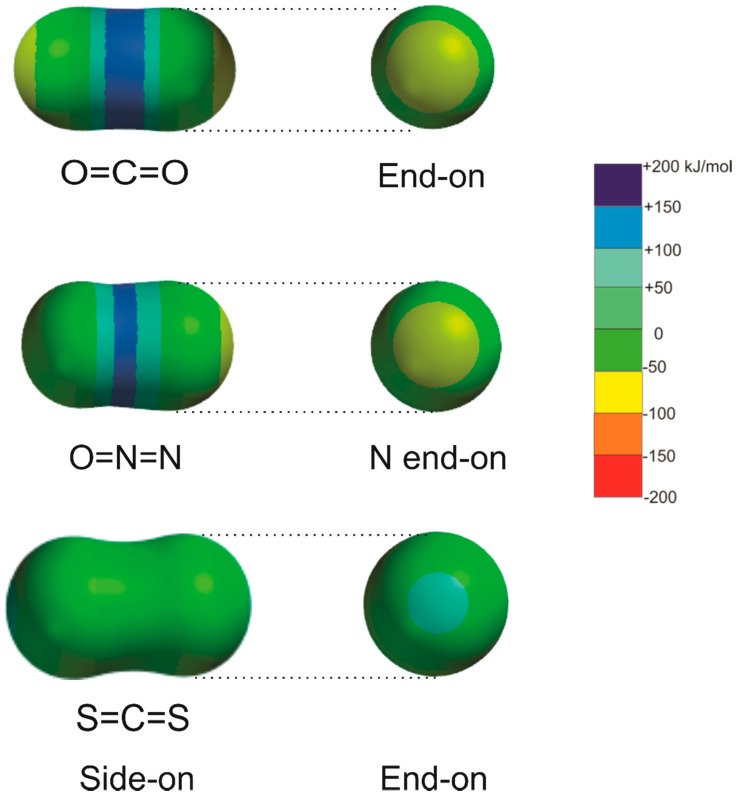
Molecular electrostatic surfaces potential (MESPs) for carbon dioxide, nitrous oxide, and carbon disulfide calculated for the 0.002 e/bohr^3^ iso-surface at the MP2/6-311++G** level.

The closely related molecules CO_2_, N_2_O, and CS_2_ form a series of interest in the context of non-covalent bonding. Each provides an electrophilic site by means of which either tetrel, pnictogen, or chalcogen bonds, respectively, could be formed. Both CO_2_ and CS_2_ are non-dipolar; thus, the molecular electric quadrupole moment is the first non-zero term in the expansion of the electric charge distribution; however, this moment is of opposite sign in the two molecules [11,12]. For CO_2_, the sign corresponds to the partial charge description ^δ−^O = ^2δ+^C = O^δ−^, while, for CS_2_, the reverse arrangement ^δ+^S = ^2δ−^C = S^δ+^ is implied. These charge distributions can be readily identified in the MESPs shown for each molecule (calculated at the 0.002 e/bohr^3^ iso-surface) in Figure 1, which shows side-on and end-on views of the MESPs of CO_2_, N_2_O, and CS_2_. Accordingly, we expect CO_2_ to form tetrel bonds perpendicular to its C_∞_ axis, via the electrophilic (blue) region at the C atom, with, e.g., the n-pair of a Lewis base. Conversely, CS_2_ is likely to form chalcogen bonds via the electrophilic (blue) region that lies at each S atom and is centered on the C_∞_ axis. Clearly, the charge distributions of CO_2_ and N_2_O, as represented by their MESPs in Figure 1, are very similar, as are the signs and magnitudes of their electric quadrupole moments [11,13]; however, N_2_O also has a small electric dipole moment. Nitrous oxide is, therefore, expected to form a complex with a given Lewis base of similar geometry to that of its carbon dioxide counterpart, but with small distortions resulting from the lower symmetry and the non-zero electric dipole moment in the case of N_2_O.

It this article, we present the geometries and interaction strengths of complexes of the type B⋯CO_2_, B⋯CS_2_, and B⋯N_2_O for the series of Lewis bases, B = CO, HCCH, H_2_S, HCN, H_2_O, PH_3_, and NH_3_, as calculated ab initio at the CCSD(T)/aug-cc-pVTZ level of theory. The geometries so calculated can be compared with those established experimentally via gas-phase rotational or vibration–rotation spectra for some, but not all, of the complexes B⋯CO_2_ [14,15,16,17,18,19,20,21] and B⋯N_2_O [21,22,23,24,25,26,27,28,29]; however, data for B⋯CS_2_ are sparse [30]. The interaction strength can be described in two possible ways. The first is the energy required for the reaction B⋯CO_2_ = B + CO_2_, that is, the equilibrium dissociation energy *D*_e_. The second is the intermolecular quadratic stretching force constant *k*_σ_, which is proportional to the energy required for a unit infinitesimal displacement from equilibrium along the dissociation coordinate. It was shown elsewhere for hydrogen-bonded complexes B⋯HX and halogen-bonded complexes B⋯XY (X and Y are halogen atoms) that *D*_e_ is directly proportional to *k*_σ_, with a constant of proportionality of 1.5(1) × 10^−3^ m^2^·mol^−1^, whether *k*_σ_ is obtained experimentally [31] from centrifugal distortion effects in the rotational spectra of the complexes or calculated ab initio [32].

Given the definitions of hydrogen and halogen bonds in terms of the interaction of nucleophilic regions of Lewis bases B with electrophilic regions near the atoms H of HX and X of XY, the aim of the work presented here is to examine by means of ab initio calculations (1) whether the complexes B⋯CO_2_, B⋯N_2_O, and B⋯CS_2_ involve tetrel, pnictogen, and chalcogen bonds, respectively, and (2) whether there is direct proportionality of *D*_e_ and *k*_σ_ for these complexes, and, if so, does the constant of proportionality found for hydrogen- and halogen-bonded complexes B⋯HX and B⋯XY also hold in these non-covalent bonds.

## 2. Theoretical Methods

**Figure 2:**
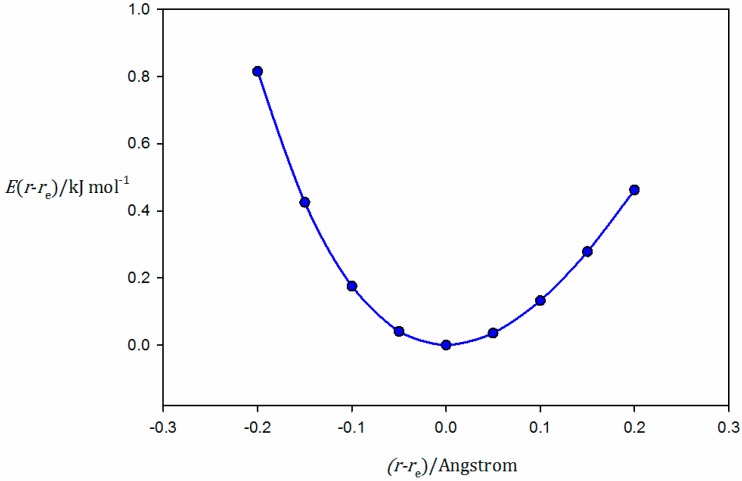
The variation in *E* (*r* − *r*_e_) with *r* − *r*_e_, used to calculate the intermolecular quadratic force *k*_σ_ (the curvature at the minimum) for H_3_N⋯S=C=S at the CCSD(T)/aug-cc-pVTZ level of theory. The curve is a third-order polynomial fit to the calculated points (*R*^2^ of fit = 0.9998). The polynomial was differentiated twice to obtain *k*_σ_.

We present here equilibrium geometries and values of *D*_e_ and $k_{\sigma }$ (defined earlier) calculated ab initio for the members of three series of complexes, namely the series of B⋯CO_2_, B⋯N_2_O, and B⋯CS_2_, where B is one of the simple Lewis bases, CO, HCCH, H_2_S, HCN, H_2_O, PH_3_, or NH_3_. The geometry optimizations and the calculations of $k_{\sigma }$ were conducted at the CCSD(T)/aug-cc-pVTZ level of theory [33,34]. To evaluate $k_{\sigma }$, the energy *E*(*r*_e_) at the equilibrium geometry was first obtained, and the energy *E*(*r*) was then scanned for ±20 pm about the appropriate equilibrium intermolecular distance *r*_e_ in increments (*r* − *r*_e_) = 5 pm with optimization in all internal coordinates but *r* at each point. The curve of *E* (*r* − *r*_e_) as a function of (*r* − *r*_e_) was fitted to a third-order polynomial in (*r* − *r*_e_), and the second derivative was evaluated at *r* = *r*_e_ to yield the quadratic force constant $k_{\sigma }={\left(\frac{\partial^{2}E\left(r\right)}{\partial r^{2}}\right)}_{r=r_{e}}$, which is the curvature at the minimum. All curves used in the evaluation of all $k_{\sigma }$ presented here are available as supplementary information, as are the optimized geometries. Figure 2 shows a plot of *E* (*r* − *r*_e_) versus (*r* − *r*_e_) for the complex H_3_N⋯S=C=S, which is predicted by the ab initio calculations to possess *C*_3v_ symmetry at equilibrium, with the linear CS_2_ molecule lying along the *C*_3_ axis of NH_3_, and therefore, with the inner S atom participating in a chalcogen bond to the n-electron pair of ammonia. Values of *D*_e_ with better accuracy were obtained using the method of extrapolation to a complete basis set [35] (CCSD(T)/CBS energy). For this purpose, the HF/aug-cc-pV*n*Z//CCSD(T)/aug-cc-pVTZ energies, with *n* = D, T, and Q, for the HF contribution and the CCSD(T)/aug-cc-pV*n*’Z//CCSD(T)/aug-cc-pVTZ, with *n*’ = T and Q, for the correlation part were obtained for each system [36]. Finally, *D*_e_ was obtained as the difference of the CCSD(T)/CBS energy of the monomers and the complex. All the ab initio calculations were performed with the MOLPRO-2012 program [37]. The Z-matrices for optimized geometries are available as supplementary information. The molecular electrostatic surface potentials were generated using of the SPARTAN electronic structure package [38] at the MP2/6-311++G** level for CO_2_, N_2_O, CS_2_, and PH_3_.

## 3. Results

### 3.1. Geometries of the B⋯CO_2_, B⋯N_2_O, and B⋯CS_2_ Complexes

**Figure 3:**
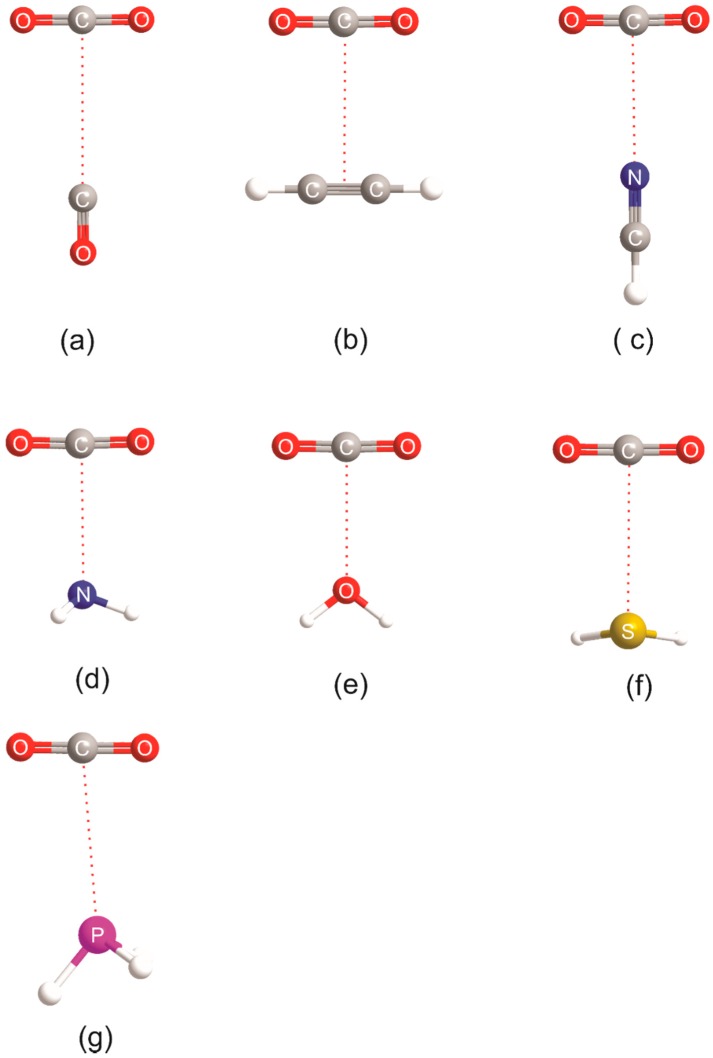
Molecular models drawn to scale of the geometries of B⋯CO_2_ complexes calculated at the CCSD(T)/aug-cc-pVTZ level of theory, where B = CO, HCCH, HCN, NH_3_, H_2_O, H_2_S, and PH_3_ (**a**–**g**, respectively). Not shown is the linear, hydrogen-bonded isomer CO_2_⋯HCN, which is 1.5 kJ·mol^−1^ higher in energy than the form in ©.

Molecular diagrams showing the equilibrium geometry (drawn to scale) of each member of the B⋯CO_2_ series, where B = CO, HCCH, H_2_S, HCN, H_2_O, PH_3_, and NH_3_, are shown in Figure 3. The calculated (equilibrium) intermolecular distances are recorded in Table 1, together with their experimental counterparts (where the latter are available). The experimental distances were determined from microwave or high-resolution infrared spectroscopy conducted on supersonically expanded gas mixtures composed of the two component molecules diluted in an inert gas. The molecular shapes and intermolecular distances are, in each case, in reasonable agreement with those from experiment. It should be noted that the experimental distances are, in most cases, of the *r*_0_ type, but are corrected for the contributions of the angular oscillations of the two components to the zero-point motion. There is no correction for the intermolecular radial contribution, however, and this normally leads to *r*_0_ distances that are greater than the calculated equilibrium values. For the very floppy molecules considered here, the *r*_0_ values are greater by the order of 0.05 to 0.1 Å.

It is clear from Figure 3 that the intermolecular bond is a tetrel bond in the sense that it involves the electrophilic region around C (the blue band that surrounds the C atom in the MESP of CO_2_ shown in Figure 1) and either a non-bonding electron pair or a π-bonding electron pair as the nucleophilic site of the Lewis base B. In fact, the axis of the non-bonding electron pair coincides with the extension of the radius of the circle that defines the most electrophilic band around C in each of OC⋯CO_2_, HCN⋯CO_2_, H_3_N⋯CO_2_, and H_2_S⋯CO_2_, given that the n-pairs on S in H_2_S lie at ~±90° to the plane of the H_2_S nuclei, as established from earlier work on H_2_S⋯HX and H_2_S⋯XY (X and Y are halogen atoms) [9,40]. The fact that the ab-initio-derived configuration at O in H_2_O⋯CO_2_ is planar is not inconsistent with this conclusion. It was found for all H_2_O⋯HX and H_2_O⋯XY [9,40] investigated through rotational spectroscopy and/or ab initio calculations that, although the equilibrium configuration at O is non-planar, the barrier to planarity is low and lies below the zero-point energy level in most cases. The configuration is, therefore, rapidly inverting in the zero-point state and the molecule is effectively planar. For an interaction as weak as that in H_2_O⋯CO_2_, the barrier will probably be non-existent, as it is in H_2_O⋯F_2_ [41], for example. Some rules put forward originally for hydrogen-bonded complexes B⋯HX [9] and halogen-bonded complexes B⋯XY [40] can be easily modified to allow the geometries of the tetrel-bonded complexes shown in Figure 3 to be predicted. Thus, the modified rules become:

*The equilibrium geometry of tetrel-bonded* B⋯CO_2_ *complexes can be predicted by assuming that a radius of the most electrophilic ring around the* C *atom of* CO_2_ *coincides with either (1) the axis of a non-bonding electron pair carried by* B*, or (2) the local symmetry axis of a π-bonding electron pair of* B.

That is, in the original rules, “hydrogen-bonded complexes B⋯HX” is replaced by “tetrel-bonded complexes B⋯CO_2_”, and “the axis of HX” is replaced by “a radius of the most electrophilic ring around the C atom of CO_2_”.

**Figure 4:**
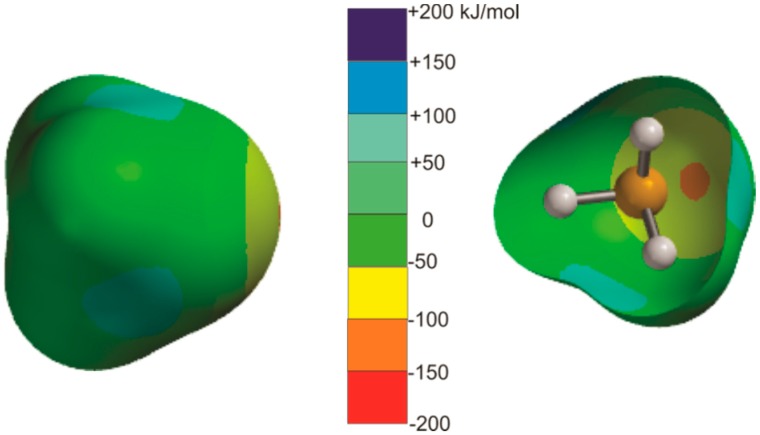
Molecular electrostatic surface potentials (MESPs) for phosphine calculated for the 0.002 e/bohr^3^ iso-surface at the MP2/6-311++G** level. The surface in the right-hand diagram is cut away to reveal both the electrophilic (blue) regions near P on approximately the extension of the H–P bonds, and the nucleophilic (red dot) region on the *C*_3_ axis.

The case of H_3_P⋯CO_2_ appears to be an exception to the rules, because the intermolecular bond does not lie exactly along the *C*_3_ axis of phosphine. The reason for this becomes clear when the MESP of phosphine, shown in Figure 4, is examined. Approximately opposite the extension of each P–H bond is an electrophilic (blue) region which can interact with the nucleophilic (yellow-green) band around O of CO_2_ (see Figure 1). This secondary interaction is, in fact, a pnictogen bond, and it is responsible for the distortion found in Figure 3g.

**Figure 5:**
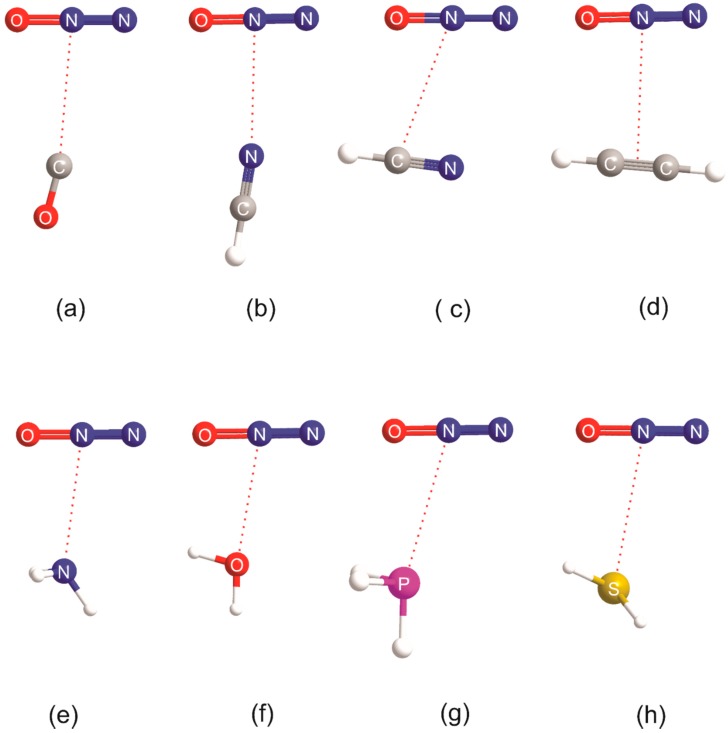
Molecular models drawn to scale of the geometries of B⋯N_2_O complexes calculated at the CCSD(T)/aug-cc-pVTZ level of theory, where B = CO, HCN, HCCH, NH_3_, H_2_O, PH_3_, and H_2_S (**a**–**h**, respectively; note that there are two models shown for HCN complexes). When B = HCN there are three low-energy conformers: the slipped parallel form at the global minimum, the T-shaped isomer higher in energy by only 0.03 kJ·mol^−1^, and a linear, hydrogen-bonded conformer N_2_O⋯HCN (not shown) higher in energy by 1.3 kJ·mol^−1^ (see text for discussion).

The molecular geometries calculated ab initio for the corresponding B⋯N_2_O series are illustrated in Figure 5, and each has a similar, but not identical, shape to that of the corresponding member of the B⋯CO_2_ series, with the central N atom of N_2_O acting as the primary electrophilic site. The lower symmetry of N_2_O compared with that of CO_2_ means, however, that the B⋯N_2_O complexes necessarily have lower symmetry and that secondary interactions become more important. The geometries shown in Figure 5 can be understood in terms of the rule set out in the preceding paragraph, that is, with the primary interaction involving the electrophilic (blue) band on the central N atom of N_2_O with the n-pair or π-pair on the Lewis base B, but modified to allow a secondary interaction of the electrophilic region of B (i.e., C or H of HCN, H of HCCH, H of NH_3_, H of H_2_O, H of PH_3_, or H of H_2_S) with the nucleophilic region at O in N_2_O (see Figure 1, end-on view).The conclusions for B⋯CO_2_ and B⋯N_2_O are, therefore, consistent with the previously noted similarity of the MESPs of CO_2_ and N_2_O displayed in Figure 1. The molecular shapes shown in Figure 5 correspond closely to those that are available experimentally (see Reference [3] for a convenient collection of experimentally determined shapes). The ab initio and experimental (where available) intermolecular distances for each B⋯N_2_O complex are included in Table 2.

Two geometries are given for HCN⋯N_2_O in Figure 5. Both correspond to minima in the energy, but are separated in energy by only 0.03 kJ·mol^−1^ at the CCSDT(T)/aug-cc-pVTZ level of theory and 0.45 kJ·mol^−1^ at the CCSD(T)/CBS level, with the parallel form (Figure 5c) lower in energy than the nearly perpendicular form (Figure 5b) in both cases. It is of interest to note that Miller and co-workers [25] found two isomers of this complex in their investigation of the high-resolution infrared spectrum of (N_2_O, HCN) in a supersonically expanded gas mixture of the components diluted in helium. One was a parallel form (four such arrangements of N_2_O and HCN were consistent with their observed rotational constants, including that found here by ab initio calculation), while the other was a hydrogen-bonded, linear isomer N=N=O⋯HCN; however, these authors did not observe the T-shaped isomer shown in Figure 5b. Our calculations at the CCSD(T)/CBS level find the linear, hydrogen-bonded form N=N=O⋯HCN to be higher in energy than the parallel isomer by 1.5 kJ·mol^−1^. This observation suggests that, while the T-shaped isomer relaxes to the parallel form in the supersonic expansion, the higher-energy, hydrogen-bonded, linear isomer does not. Both linear, hydrogen-bonded [39,42] and T-shaped, tetrel-bonded [15,16] isomers of (CO_2_, HCN) were observed experimentally. At the CCSD(T)/CBS level, O=C=O⋯HCN is found to be 1.3 kJ·mol^−1^ higher in energy than the T-shaped isomer, in agreement with the experimental conclusions.

**Figure 6:**
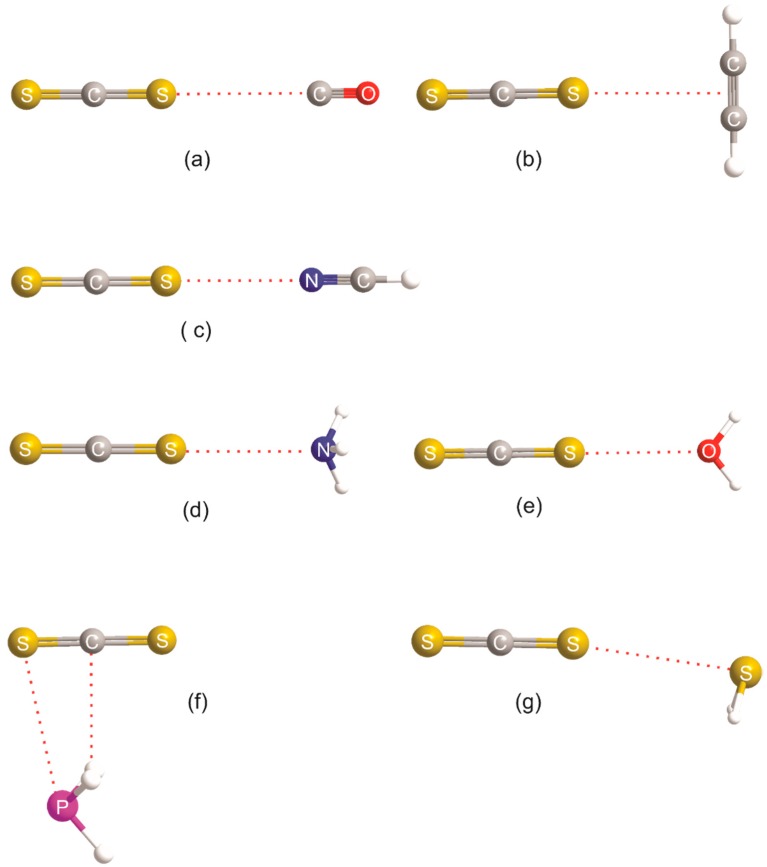
Molecular models drawn to scale of the geometries of B⋯CS_2_ complexes calculated at the CCSD(T)/aug-cc-pVTZ level of theory, where B = CO, HCCH, HCN, NH_3_, H_2_O, PH_3_ and H_2_S (**a**–**g**, respectively).

We emphasized in the introduction that the MESP of carbon disulfide is different from those of CO_2_ and N_2_O in that the most electrophilic (blue) site of CS_2_ lies on the C_∞_ axis at the surface of each S atom (see Figure 1). As is clear from Figure 6, which displays the geometries of seven B⋯CS_2_ complexes calculated at the CCSD(T)/cc-aug-pVTZ level of theory, all complexes but H_3_P⋯CS_2_ do indeed involve a chalcogen bond formed by the axial electrophilic region at one of the S atoms of CS_2_ with an n- or π-electron pair of the Lewis base B. The calculated intermolecular distances are collected in Table 3. To the best of our knowledge, only H_2_O⋯CS_2_ was investigated by means of its rotational spectrum [30]. The resulting value of *r*(O⋯S) is included in Table 3. The angular geometries of the B⋯CS_2_ complexes displayed in Figure 6 can also be predicted by the rules set out elsewhere for hydrogen-bonded complexes B⋯HX [9] or halogen-bonded complexes B⋯XY [40], if they are modified by replacing, for example, “hydrogen-bonded complexes B⋯HX” by “chalcogen-bonded complexes B⋯CS_2_” and the “HX axis” by “C_∞_ axis of CS_2_” in the wording (see earlier). We note that there is a planar configuration at O found theoretically (see Figure 6) and experimentally [30] for H_2_O⋯CS_2_, rather than the pyramidal configuration predicted by the rules. The explanation for this difference is identical to that given earlier for H_2_O⋯CO_2_. On the other hand, the configuration at S in H_2_S⋯CS_2_ is strongly pyramidal, with the intermolecular bond making an angle of approximately 90° with the plane of the H_2_S nuclei, as found for almost all H_2_S⋯HX and H_2_S⋯XY complexes so far investigated [40]. However, there is a significant non-linearity of the S⋯S=C nuclei. A possible reason for this non-linearity is that the intermolecular bond is very weak (*D*_e_ = 5.28 kJ·mol^−1^_,_ see Section 3.2) and the pair of equivalent electrophilic H atoms can undergo a secondary interaction with the weakly nucleophilic (yellow-green) region of CS_2_ (see the MESP of CS_2_ in Figure 1). The geometry of H_3_P⋯CS_2_ involves a pnictogen bond and can be understood by reference to the MESP of phosphine in Figure 4. It seems that the primary interaction here involves one of the electrophilic (blue) regions near to P and approximately on the extension of each P–H bond (as seen in the cutaway version of the phosphine MESP in Figure 4) with the nucleophilic (yellow-green) region of CS_2_. Evidently, this interaction is stronger than that of the terminal electrophilic (blue) region at S with the n-electron pair of phosphine (the red spot in the cutaway version of the MESP in Figure 4), leading to a primary P pnictogen bond.

### 3.2. The Relationship between D_e_ and k_σ_ in the B⋯CO_2_, B⋯N_2_O, and B⋯CS_2_ Series

It was established [31] for a wide range of hydrogen-bonded complexes B⋯HX (X = F, Cl, Br, or I) and halogen-bonded complexes B⋯XY (X and Y are halogen atoms) that their dissociation energies *D*_e_ (as calculated ab initio at the CCSD(T)(F12c)/cc-pvdz-F12 level of theory) are directly proportional to their intermolecular stretching force constants *k*_σ_ (as determined experimentally from centrifugal distortion constants *D_J_* or *Δ_J_* obtained by measuring rotational spectra). The constant of proportionality was found to be 1.5(1) × 10^3^ m^2^·mol^−1^. Later, it was shown for the B⋯HF, B⋯HCl, B⋯F_2_, B⋯Cl_2_, and B⋯ClF series, where B is a Lewis base, N_2_, CO, HCCH, C_2_H_4_, HCN, H_2_S, H_2_O, PH_3_, or NH_3_, that the same constant of proportionality applies [32] when *k*_σ_ was calculated ab initio at the CCSD(T)/aug-cc-pVTZ level of theory and *D*_e_ was obtained via a CCSD(T)/CBS calculation, where CBS indicates a complete basis-set extrapolation using the aug-cc-pV*n*Z (*n* = T and Q) basis sets. The opportunity is taken here to investigate the corresponding relationship for the tetrel-bonded B⋯CO_2_ complexes, the pnictogen-bonded B⋯N_2_O complexes, and the chalcogen-bonded B⋯CS_2_ complexes for the series of Lewis bases, B = CO, HCCH, H_2_S, HCN, H_2_O, PH_3_, and NH_3_, when both *k*_σ_ and *D*_e_ are calculated in the same way as described in Reference [32].

**Figure 7:**
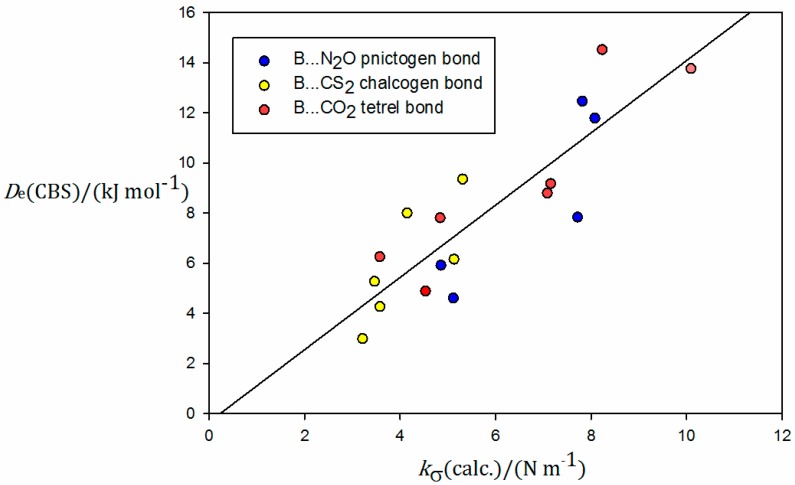
Plot of *D*_e_ calculated at the CCSD(T)/CBS level of theory (CBS indicates a complete basis-set extrapolation using the aug-cc-pV*n*Z (*n* = T and Q) basis sets) versus *k*_σ_ calculated at the CCSD(T)/aug-cc-pVTZ level for B⋯CO_2_, B⋯N_2_O, and B⋯CS_2_ complexes. See text for discussion.

**Figure 8:**
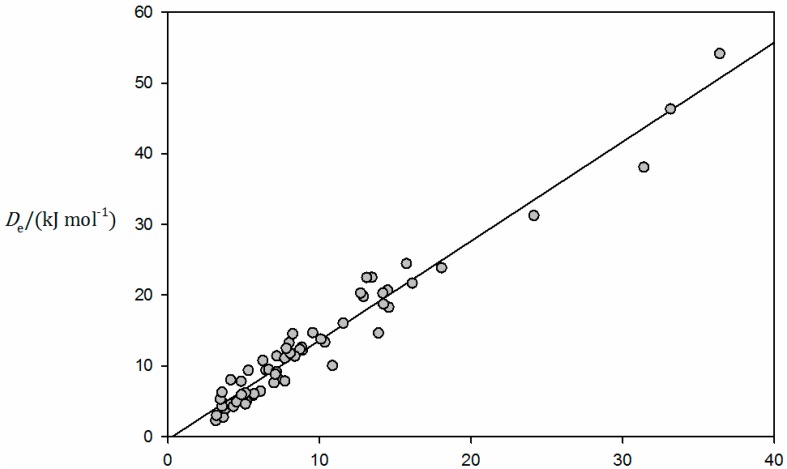
Plot of *D*_e_ calculated at the CCSD(T)/CBS level versus *k*_σ_ calculated at the CCSD(T)/aug-cc-pVTZ level for B⋯CO_2_, B⋯N_2_O, and B⋯CS_2_ complexes (this work; see also Figure 7), and B⋯HF, B⋯HCl, B⋯F_2_, B⋯Cl_2_, and B⋯ClF complexes (see Reference [32] for the Lewis bases B involved and the values of *D*_e_ and *k*_σ_ for the B⋯HX and B⋯XY series).

Values of *D*_e_ and *k*_σ_ so determined are recorded in Table 4, while Figure 7 shows a plot of *D*_e_ as the ordinate and *k*_σ_ as the abscissa for the B⋯CO_2_, B⋯N_2_O, and B⋯CS_2_ series investigated here, with color coding of the points as red, blue, and yellow, respectively. For consistency with HCN⋯CO_2_, of the isomers of HCN⋯N_2_O, only the data for the T-shaped form are included in Table 4 and Figure 7. The calculation of *k*_σ_ for the parallel isomer of N_2_O⋯HCCH was prevented by convergence problems, as well as for H_2_S⋯N_2_O because, as the N⋯S distance was varied, there was a switch to the hydrogen-bonded arrangement N_2_O⋯HSH. H_3_P⋯CS_2_ was excluded because it does not involve a chalcogen bond, unlike the remaining B⋯CS_2_ complexes. The results of a linear regression fit of the points in Figure 7 are as follows: gradient = 1.44(20) × 10^3^ m^2^·mol^−1^ and intercept on the ordinate = −0.32(124) kJ·mol^−1^. Thus, within the errors of the fit, *D*_e_ and *k*_σ_ are directly proportional, and the slope of the regression line agrees with those found previously for the B⋯HF and B⋯HCl series, and for the halogen-bonded series B⋯F_2_, B⋯Cl_2_, and B⋯ClF [32] when calculations were conducted at identical levels of theory, namely 1.38(7) × 10^3^ m^2^·mol^−1^ and 1.49(5) × 10^3^ m^2^·mol^−1^, respectively. Plots of *D*_e_ versus *k*_σ_ using *D*_e_ values calculated at the CCSD(T)(F12c)/cc-pVDZ-F12 level of theory and experimentally available *k*_σ_ [31], but with many more complexes in each of these two classes, gave almost identical slopes of 1.52(3) × 10^3^ m^2^·mol^−1^ and 1.47(3) × 10^3^ m^2^·mol^−1^, respectively. Evidently, the same relationship between *D*_e_ and *k*_σ_ holds for hydrogen-bonded complexes B⋯HX, halogen-bonded complexes B⋯XY, the tetrel-bonded complexes B⋯CO_2_, the pnictogen-bonded complexes B⋯N_2_O, and the chalcogen-bonded complexes B⋯CS_2_. This fact is visually established by the plot of *D*_e_ versus *k*_σ_ shown in Figure 8. The figure includes all B⋯HF, B⋯HCl, B⋯F_2_, B⋯Cl_2_, and B⋯ClF complexes reported in Reference [32] and all the B⋯CO_2_, B⋯N_2_O, and B⋯CS_2_ complexes included in Figure 7. Both sets of series were calculated in the same way, i.e., CCSD(T)/aug-cc-pVTZ for *k*_σ_ and CCSD(T)/CBS for *D*_e_. The linear regression fit for all these data leads to 1.40(4) × 10^3^ m^2^·mol^−1^ for the slope and −0.42(46) kJ·mol^−1^ for the intercept.

## 4. Conclusions

The series of B⋯CO_2_, B⋯N_2_O, and B⋯CS_2_ complexes was investigated through ab initio calculations at the CCSD(T)/aug-pVTZ level of theory for the Lewis bases, B = CO, HCCH, H_2_S, HCN, H_2_O, PH_3_, and NH_3_. The atoms, except for some H, lie in a plane for all complexes. The intermolecular bonds in the B⋯CO_2_ complexes are formed by interaction of the electrophilic region around the C atom of CO_2_ (see Figure 1) with n- or π-electron pairs (nucleophilic regions) carried by B and are, therefore, tetrel bonds. The geometry of each B⋯N_2_O complex investigated (except perhaps for B = PH_3_) is similar to that of the corresponding member of the B⋯CO_2_ series. Thus, the primary non-covalent interaction involves the central N atom of N_2_O with an n- or π-electron pair carried by B, but moderated by distortions that appear to arise from the secondary interaction of the electrophilic region of B (e.g., H atoms) with the O atom of N_2_O. The B⋯CS_2_ series is geometrically distinct from the other two in that (apart from B = PH_3_) the primary non-covalent interaction is between the electrophilic region centered on the C_∞_ axis of CS_2_ near to an S atom (see Figure 1) and an n- or π-electron pair of B, leading to a linear (or nearly linear in the case of B = H_2_S) C=S⋯B system, and is, therefore, a chalcogen bond. These interpretations are electrostatic in origin and were applied previously to hydrogen bonds in B⋯HX complexes [9] and halogen bonds in B⋯XY complexes [40]. Consistent with the foregoing observations is the fact that the geometries of members of each of the three series, B⋯CO_2_, B⋯N_2_O, and B⋯CS_2_, can be predicted by rules put forward some years ago for the same purpose for hydrogen-bonded complexes B⋯HX and halogen-bonded complexes B⋯XY. Moreover, this close relationship between hydrogen, halogen, tetrel, pnictogen, and chalcogen bonds is reflected in the recent generalized definition [43] proposed for non-covalent (E) bonds based on electrostatics, provided below.

*An* E *bond occurs when there is evidence of a net attractive interaction between an electrophilic region associated with an* E *atom in a molecular entity and a nucleophilic region (e.g., an n-pair or π-pair of electrons) in another, or the same, molecular entity, where* E *is the general name for an element of Group 1, 11, 14, 15, 16, or 17 in the Periodic Table.*

We note that some complexes investigated here can be described as of the σ-hole type, while others belong to the π-hole type.

Finally, we showed that the similarity between all of these types of non-covalent interaction extends to the direct proportionality of the dissociation energy *D*_e_ and the quadratic intermolecular stretching force constant *k*_σ_, with a constant of proportionality 1.45(7) × 10^3^ m^2^·mol^−1^ describing all the series, B⋯HF, B⋯HCl, B⋯F_2_, B⋯Cl_2_, B⋯ClF, B⋯CO_2_, B⋯N_2_O, and B⋯CS_2_, when the two measures of binding strength are calculated at the CCSD(T)/CBS and CCSD(T)/aug-cc-pVTZ levels of theory, respectively. As discussed in Reference [31], a Morse function is an example of a potential energy curve for which the dissociation energy and the force constant are directly proportional.

## Figures and Tables

**Table 1:** Calculated and observed intermolecular distances in B⋯CO_2_ complexes.

Complex	Intermolecular Distance/Å		(Obs. − Calc.)/Å
	Calculated Ab Initio *^a^*	Observed	
OC⋯CO_2_	*r*(C⋯C) = 3.189	3.277(1) *^b^*	0.088(1)
HCCH⋯CO_2_	*r*(π _center_⋯C) = 3.201	3.285(3) *^c^*	0.084(3)
HCN⋯CO_2_ (T-shaped)	*r*(N⋯C) = 2.962	2.99(2) *^d^*	0.03(2)
CO_2_⋯HCN (linear)	*r*(O⋯H) = 2.236	2.34 *^e^*	0.11
H_3_N⋯CO_2_	*r*(N⋯C) = 2.922	2.9875(2) *^f^*	0.066
H_2_O⋯CO_2_	*r*(O⋯C) = 2.758	2.836 *^g^*	0.078
H_2_S⋯CO_2_	*r*(S⋯C) = 3.425	3.449(1) *^h^*	0.024(1)
H_3_P⋯CO_2_	*r*(P⋯C) = 3.528	⋯	⋯

*^a^* See Figure 3 for the molecular diagrams (to scale) of the B⋯CO_2_ complexes. *^b^* Reference [14]; *^c^* Reference [17]; *^d^* Reference [15,16]. *^e^* The distance reported here is the *r*_s_ value from Reference [39]; *^f^* Reference [20]; *^g^* Reference [19]; *^h^* Reference [18].

**Table 2:** Calculated and observed intermolecular distances in B⋯N_2_O complexes.

Complex	Intermolecular Distance/Å		(Obs. − Calc.)/Å
	Calculated Ab Initio *^a^*	Observed	
OC⋯N_2_O	*r*(C⋯N_center_) = 3.176	3.36(1) *^b^*	0.18
HCCH⋯N_2_O	*r*(π_center_⋯N_center_) = 3.201	3.296 *^c^*	0.095(1)
HCN⋯N_2_O (T-shaped)	*r*(C⋯N_center_) = 3.002	⋯	⋯
HCN⋯N_2_O (parallel)	*r*(C⋯N_center_) = 3.271	3.392 *^d^*	0.121
H_3_N⋯N_2_O	*r*(N⋯N_center_) = 3.021	3.088 *^e^*	0.067
H_2_O⋯N_2_O	*r*(O⋯N_center_) = 2.855	2.97(2) *^f^*	0.11(2)
H_2_S⋯N_2_O	*r*(S⋯N_center_) = 3.444	⋯	⋯
H_3_P⋯N_2_O	*r*(P⋯N_center_) = 3.479	⋯	⋯

*^a^* See later for the molecular diagrams (to scale) of the B⋯N_2_O complexes. *^b^* *r*_s_ value estimated from data in Reference [24] is almost certainly an overestimate, as *b*_N_ is very small, and therefore, severely underestimated. *^c^* References [26,27]; *^d^* Reference [25]; *^e^* Reference [29]; *^f^* Reference [28].

**Table 3:** Calculated and observed intermolecular distances in B⋯CS_2_ complexes.

Complex	Intermolecular Distance/Å		(Obs. − Calc.)/Å
	Calculated Ab Initio *^a^*	Observed	
OC⋯CS_2_	*r*(C⋯S) = 3.616	⋯	⋯
HCCH⋯CS_2_	*r*(π_center_⋯S) = 3.568	⋯	⋯
HCN⋯CS_2_	*r*(N⋯S) = 3.285	⋯	⋯
H_3_N⋯CS_2_	*r*(N⋯S) = 3.304	⋯	⋯
H_2_O⋯CS_2_	*r*(O⋯S) = 3.132	3.197 *^b^*	0.065
H_2_S⋯CS_2_	*r*(S⋯S) = 3.773	⋯	⋯
H_3_P⋯CS_2_	*r*(P⋯S) = 3.798	⋯	⋯

*^a^* See Figure 6 for the molecular diagrams (to scale) of the B⋯CS_2_ complexes. *^b^* Reference [30].

**Table 4:** Intermolecular dissociation energies *D*_e_ and quadratic force constants *k*_σ_ for B⋯CO_2_, B⋯N_2_O, and B⋯CS_2_ complexes.

Lewis Base B	B⋯CO_2_		B⋯N_2_O		B⋯CS_2_	
	*D*_e_/kJ·mol^−1^	*k*_σ_/(N·m^−1)^	*D*_e_/kJ·mol^−1^	*k*_σ_/(N·m^−1)^	*D*_e_/kJ·mol^−1^	*k*_σ_/(N·m^−1)^
OC	4.89	4.53	4.61	5.13	2.99	3.21
HCCH	8.81	7.08	8.14	⋯ *^a^*	4.27	3.58
HCN	9.18	7.15	7.84	7.72	6.16	5.13
H_2_O	13.77	10.09	12.47	7.82	8.01	4.15
H_2_S	7.82	4.84	7.25	⋯ *^b^*	5.28	3.46
H_3_N	14.53	8.23	11.79	8.08	9.36	5.31
H_3_P	6.26	4.85	5.92	4.85	6.75	⋯ *^c^*

*^a^* Convergence problems when attempting to calculate the *E* (*r* − *r*_e_) versus (*r* − *r*_e_) curve to obtain *k*_σ_. *^b^* When attempting to calculate *k*_σ_, the geometry of the complex changes to the hydrogen-bonded isomer N_2_O⋯HSH as (*r* − *r*_e_) increases. *^c^* The main non-covalent interaction in this complex is between P of PH_3_ and S of CS_2_, and it is a pnictogen bond, not a chalcogen bond.

## Supplementary Materials

The supplementary materials are available.
