# Supplementary material for: An Ab Initio Investigation of the Geometries and Binding Strengths of Tetrel-, Pnictogen-, and Chalcogen-Bonded Complexes of CO2, N2O, and CS2 with Simple Lewis Bases: Some Generalizations
Source: Molecules. 2018 Sep 4;23(9):2250. doi: 10.3390/molecules23092250 (PMC6225110; doi:10.3390/molecules23092250)
Supplement: Supplementary file 1 [file molecules-23-02250-s001.zip › molecules-352721-supplementary-original/SI_1z_matrix-geoms.pdf]

CO_2	C<br>X,1,1.<br>O,1,r1,2,90.<br>O,1,r1,2,90.,3,180.,0<br><br>R1= 1.16699443 ANGSTROM
CO_2:CO	C<br>X,1,1.<br>O,1,r1,2,a1<br>O,1,r1,2,a1,3,180.,0<br>C,1,r2,3,a1,2,0.,0<br>O,1,r3,3,a1,2,0.,0<br><br>R1= 1.16689562 ANGSTROM<br>A1= 90.26092656 DEGREE<br>R2= 3.18895171 ANGSTROM<br>R3= 4.32419001 ANGSTROM
CO_2:HCCH	C<br>X,1,1.<br>O,1,r1,2,a1<br>O,1,r1,2,a1,3,180.,0<br>X,1,r2,3,a1,2,0.,0<br>C,5,r3,2,90.,3,0.,0<br>C,5,r3,2,90.,4,0.,0<br>H,5,r4,2,a4,3,0.,0<br>H,5,r4,2,a4,4,0.,0<br><br>R1= 1.16695767 ANGSTROM<br>A1= 90.39127333 DEGREE<br>R2= 3.20093589 ANGSTROM<br>R3= 0.60538158 ANGSTROM<br>R4= 1.66986445 ANGSTROM<br>A4= 89.81599345 DEGREE
CO_2:NCH(T- shaped)	C<br>X,1,1.<br>O,1,r1,2,a1<br>O,1,r1,2,a1,3,180.,0<br>N,1,r2,3,a1,2,0.,0<br>C,1,r3,3,a1,2,0.,0<br>H,1,r4,3,a1,2,0.,0<br><br>R1= 1.16688463 ANGSTROM<br>A1= 90.76445447 DEGREE<br>R2= 2.96202581 ANGSTROM<br>R3= 4.12143255 ANGSTROM<br>R4= 5.18937839 ANGSTROM

CO2-HCN (linear, H-bonded)	C<br>X,1,1.<br>O,1,R1,2,90.<br>O,1,R2,2,90.,3,180.,0<br>H,1,R3,2,90.,3,180.,0<br>X,5,1.,1,90.,2,0.,0<br>C,5,R4,6,90.,1,180.,0<br>N,5,R5,6,90.,1,180.,0<br><br>R1= 1.16368133 ANGSTROM<br>R2= 1.16904557 ANGSTROM<br>R3= 3.39974308 ANGSTROM<br>R4= 1.06848510 ANGSTROM<br>R5= 2.22872961 ANGSTROM<br><br>CCSD(T)/CBS energy= -281.741349260795 Hartree (1.3 kJ/mol less stable than the T shaped structure)
CO_2:NH_3	C<br>X,1,1.<br>O,1,r1,2,90.<br>O,1,r2,2,a1,3,180.,0<br>N,1,r3,3,a3,2,0.,0<br>H,5,r4,1,a4,3,180.,0<br>H,5,r5,1,a5,3,d5,0<br>H,5,r5,1,a5,3,-d5,0<br><br>R1= 1.16703785 ANGSTROM<br>R2= 1.16719581 ANGSTROM<br>A1= 92.59741743 DEGREE<br>R3= 2.92218508 ANGSTROM<br>A3= 91.62419041 DEGREE<br>R4= 1.01517012 ANGSTROM<br>A4= 110.97260314 DEGREE<br>R5= 1.01524170 ANGSTROM<br>A5= 113.09898728 DEGREE<br>D5= 60.39689047 DEGREE
CO_2:OH_2	C<br>X,1,1.<br>O,1,r1,2,a1<br>O,1,r1,2,a1,3,180.,0<br>O,1,r2,3,a1,2,0.,0<br>H,5,r3,1,a3,3,0.,0

	H,5,r3,1,a3,4,0.,0<br><br>R1= 1.16688256 ANGSTROM<br>A1= 90.94990727 DEGREE<br>R2= 2.75775306 ANGSTROM<br>R3= 0.96167110 ANGSTROM<br>A3= 127.62334371 DEGREE
CO_2:PH_3	C<br>X,1,1.<br>O,1,r1,2,90.<br>O,1,r2,2,a1,3,180.,0<br>P,1,r3,2,a3,3,0.,0<br>H,5,r4,1,a4,3,180.,0<br>H,5,r5,1,a5,3,d5,0<br>H,5,r5,1,a5,3,-d5,0<br><br>R1= 1.16744332 ANGSTROM<br>R2= 1.16664608 ANGSTROM<br>A1= 90.78332173 DEGREE<br>R3= 3.52758267 ANGSTROM<br>A3= 4.49836564 DEGREE<br>R4= 1.41846385 ANGSTROM<br>A4= 136.64671037 DEGREE<br>R5= 1.41870649 ANGSTROM<br>A5= 114.69537750 DEGREE<br>D5= 53.45250399 DEGREE
CO_2:SH_2	C<br>X,1,1.<br>O,1,r1,2,a1<br>O,1,r1,2,a1,3,180.,0<br>S,1,r2,3,a1,2,0.,0<br>H,5,r3,1,a3,3,d3,0<br>H,5,r3,1,a3,4,-d3,0<br><br>R1= 1.16703609 ANGSTROM<br>A1= 90.47410791 DEGREE<br>R2= 3.42489299 ANGSTROM<br>R3= 1.34213053 ANGSTROM<br>A3= 96.11653785 DEGREE<br>D3= 43.40684932 DEGREE

N_2O	N<br>X 1 1.<br>N 1 r1 2 90.<br>O 1 r2 2 90. 3 180.<br><br>R1= 1.13266309 ANGSTROM<br>R2= 1.19119506 ANGSTROM
N_2O:CO	N<br>X 1 1.<br>N 1 r1 2 90.<br>O 1 r2 2 a2 3 180.<br>C 1 r3 3 a3 2 180.<br>X 5 1. 1 90. 3 0.<br>O 5 r4 6 a4 1 180.<br><br>R1= 1.13210638 ANGSTROM<br>R2= 1.19095036 ANGSTROM<br>A2= 90.12864491 DEGREE<br>R3= 3.17630540 ANGSTROM<br>A3= 94.90525654 DEGREE<br>R4= 1.13540183 ANGSTROM<br>A4= 99.78237333 DEGREE
N_2O:HCCH	N<br>X 1 1.<br>N 1 B2 2 90.<br>O 1 B3 2 A2 3 180.<br>X 1 B4 3 A3 2 0.<br>C 5 B5 1 A4 3 0.<br>C 5 B5 1 A5 4 0.<br>H 5 B7 1 A6 3 0.<br>H 5 B8 1 A7 4 0.<br><br>B2= 1.13191032 ANGSTROM<br>B3= 1.19074773 ANGSTROM<br>A2= 89.80799318 DEGREE<br>B4= 3.22197129 ANGSTROM<br>A3= 92.38294855 DEGREE<br>B5= 0.60539272 ANGSTROM<br>A4= 93.04456666 DEGREE<br>A5= 86.24445372 DEGREE<br>B7= 1.66979961 ANGSTROM<br>A6= 93.34146896 DEGREE<br>B8= 1.66974609 ANGSTROM<br>A7= 86.18314121 DEGREE
N_2O:NCH	N<br>X 1 1.<br>N 1 r1 2 90.<br>O 1 r2 2 a2 3 180.<br>N 1 r3 3 a3 2 180.

	X 5 1. 1 90. 3 0.<br>C 5 r4 6 a4 1 180.<br>H 5 r5 6 a5 1 180.<br><br>R1= 1.13178957 ANGSTROM<br>R2= 1.19055736 ANGSTROM<br>A2= 89.70319317 DEGREE<br>R3= 3.00237128 ANGSTROM<br>A3= 92.51860891 DEGREE<br>R4= 1.15957570 ANGSTROM<br>A4= 97.69934838 DEGREE<br>R5= 2.22743057 ANGSTROM<br>A5= 97.72652993 DEGREE
$N_2O:OH_2$	N<br>X 1 1.<br>N 1 r1 2 90.<br>O 1 r2 2 a2 3 180.<br>O 1 r3 3 a3 2 180.<br>H 5 r4 1 a4 3 0.<br>H 5 r5 1 a5 4 0.<br><br>R1= 1.13038009 ANGSTROM<br>R2= 1.19356294 ANGSTROM<br>A2= 89.58216709 DEGREE<br>R3= 2.85478673 ANGSTROM<br>A3= 100.63604826 DEGREE<br>R4= 0.96132692 ANGSTROM<br>A4= 171.06312463 DEGREE<br>R5= 0.96229856 ANGSTROM<br>A5= 84.16208585 DEGREE
$N_2O:SH_2$	N<br>X 1 1.<br>N 1 r1 2 90.<br>O 1 r2 2 a2 3 180.<br>S 1 r3 3 a3 2 180.<br>H 5 r4 1 a4 3 d4<br>H 5 r5 1 a5 4 d5<br><br>R1= 1.13165091 ANGSTROM<br>R2= 1.19211561 ANGSTROM<br>A2= 90.03095303 DEGREE<br>R3= 3.44374168 ANGSTROM<br>A3= 100.17649793 DEGREE<br>R4= 1.34202505 ANGSTROM<br>A4= 116.84829282 DEGREE<br>D4= 54.89834523 DEGREE<br>R5= 1.34199764 ANGSTROM<br>A5= 79.01178960 DEGREE<br>D5= -37.76856958 DEGREE
$N_2O:NH_3$	N

	X 1 1.<br>N 1 r1 2 90.<br>O 1 r2 2 a2 3 180.<br>N 1 r3 3 a3 2 180.<br>H 5 r4 1 a4 3 0.<br>H 5 r5 1 a5 4 d5<br>H 5 r5 1 a5 4 -d5<br><br>R1= 1.13098877 ANGSTROM<br>R2= 1.19179889 ANGSTROM<br>A2= 89.55023730 DEGREE<br>R3= 3.02111852 ANGSTROM<br>A3= 97.84862436 DEGREE<br>R4= 1.01499854 ANGSTROM<br>A4= 137.70190324 DEGREE<br>R5= 1.01534736 ANGSTROM<br>A5= 98.49788997 DEGREE<br>D5= 53.86846339 DEGREE
$N_2O:PH_3$	N<br>X 1 1.<br>N 1 r1 2 90.<br>O 1 r2 2 a2 3 180.<br>P 1 r3 3 a3 2 180.<br>H 5 r4 1 a4 3 180.<br>H 5 r5 1 a5 4 d5<br>H 5 r5 1 a5 4 -d5<br><br>R1= 1.13171135 ANGSTROM<br>R2= 1.19246557 ANGSTROM<br>A2= 90.14148022 DEGREE<br>R3= 3.47922869 ANGSTROM<br>A3= 106.98783124 DEGREE<br>R4= 1.41889443 ANGSTROM<br>A4= 194.97926109 DEGREE<br>R5= 1.41867129 ANGSTROM<br>A5= 96.33357103 DEGREE<br>D5= 47.21461949 DEGREE

CS_2	C<br>X,1,1.<br>S,1,r1,2,90.<br>S,1,r1,2,90.,3,180.,0 }<br><br>R1= 1.56547025 ANGSTROM
CS_2:CO	C<br>X,1,1.<br>S,1,r1,2,90.<br>S,1,r2,2,90.,3,180.,0<br>C,1,r3,2,90.,4,0.,0<br>O,1,r4,2,90.,4,0.,0<br><br>R1= 1.56626815 ANGSTROM<br>R2= 1.56588685 ANGSTROM<br>R3= 5.08860843 ANGSTROM<br>R4= 6.22423311 ANGSTROM
CS_2:HCCH	C<br>X,1,1.<br>S,1,r1,2,90.<br>S,1,r2,2,90.,3,180.,0<br>X,1,r3,2,90.,4,0.,0<br>C,5,r4,1,90.,2,0.,0<br>C,5,r4,1,90.,2,180.,0<br>H,5,r5,1,a5,2,0.,0<br>H,5,r5,1,a5,2,180.,0<br><br>R1= 1.56672045 ANGSTROM<br>R2= 1.56590668 ANGSTROM<br>R3= 5.13418872 ANGSTROM<br>R4= 0.60526361 ANGSTROM<br>R5= 1.66965346 ANGSTROM<br>A5= 90.01235305 DEGREE
CS_2:NCH	C<br>X,1,1.<br>S,1,r1,2,90.<br>S,1,r2,2,90.,3,180.,0<br>N,1,r3,2,90.,4,0.,0<br>C,1,r4,2,90.,4,0.,0<br>H,1,r5,2,90.,4,0.,0<br><br>R1= 1.56903001 ANGSTROM<br>R2= 1.56449571 ANGSTROM<br>R3= 4.84933103 ANGSTROM<br>R4= 6.00908780 ANGSTROM<br>R5= 7.07712244 ANGSTROM
CS_2:NH_3	C<br>X,1,1.<br>S,1,r1,2,90.

	S,1,r2,2,90.,3,180.,0<br>N,1,r3,2,90.,4,0.,0<br>H,5,r4,1,a4,2,0.,0<br>H,5,r4,1,a4,2,120.,0<br>H,5,r4,1,a4,2,-120.,0<br><br>R1= 1.56927998 ANGSTROM<br>R2= 1.56557077 ANGSTROM<br>R3= 4.86960043 ANGSTROM<br>R4= 1.01526978 ANGSTROM<br>A4= 112.46411542 DEGREE
CS_2:OH_2	C<br>X,1,1.<br>S,1,r1,2,90.<br>S,1,r2,2,90.,3,180.<br>O,1,r3,2,90.,4,0.<br>H,5,r4,1,a4,2,0.<br>H,5,r4,1,a4,2,180.<br><br>R1= 1.56908753 ANGSTROM<br>R2= 1.56474300 ANGSTROM<br>R3= 4.69698267 ANGSTROM<br>R4= 0.96186448 ANGSTROM<br>A4= 127.83925153 DEGREE
CS_2:PH_3	C<br>X,1,1.<br>S,1,r1,2,90.<br>S,1,r2,2,a1,3,180.,0<br>P,1,r3,2,a3,3,0.,0<br>H,5,r4,1,a4,3,180.,0<br>H,5,r5,1,a5,3,d5,0<br>H,5,r5,1,a5,3,-d5,0<br><br>R1= 1.56556725 ANGSTROM<br>R2= 1.56539052 ANGSTROM<br>A1= 89.42702346 DEGREE<br>R3= 3.79373352 ANGSTROM<br>A3= 11.75237846 DEGREE<br>R4= 1.42033793 ANGSTROM<br>A4= 128.90473280 DEGREE<br>R5= 1.41988053 ANGSTROM<br>A5= 55.17376969 DEGREE<br>D5= 117.62108257 DEGREE
CS_2:SH_2	C<br>X,1,1.<br>S,1,r1,2,90.<br>S,1,r2,2,90.,3,180.,0<br>S,1,r3,2,a3,4,0.,0<br>H,5,r4,1,a4,2,d4,0<br>H,5,r4,1,a4,2,-d4,0

	R1= 1.56670591 ANGSTROM<br>R2= 1.56608708 ANGSTROM<br>R3= 5.32695751 ANGSTROM<br>A3= 95.98545593 DEGREE<br>R4= 1.34227372 ANGSTROM<br>A4= 83.64947707 DEGREE<br>D4= 133.48813863 DEGREE
